# Late-Presenting Myocardial Infarction With Separate Anomalous Right-Sided Origins of the Left Anterior Descending and Circumflex Arteries: A Case Report

**DOI:** 10.7759/cureus.118026

**Published:** 2026-10-16

**Authors:** Wassim Beladel, Youssef Zeryouh, Mohamed El Minaoui

**Affiliations:** 1 Cardiology, Agadir University Center, Agadir, MAR; 2 Cardiology, Centre Hospitalier Universitaire Mohammed VI d'Agadir, Agadir, MAR; 3 Cardiology, Souss-Massa University Hospital, Faculty of Medicine and Pharmacy of Agadir, Ibn Zohr University, Agadir, MAR

**Keywords:** anomalous aortic origin of a coronary artery, case report, coronary computed tomography angiography, myocardial infarction, percutaneous coronary intervention

## Abstract

The clinical significance of an anomalous aortic origin of a coronary artery depends on the proximal course and ostial morphology, not on the ectopic origin alone, and coexisting acute myocardial infarction and multivessel atherosclerotic disease can make culprit assignment and revascularization difficult. A 79-year-old man was diagnosed with a late-presenting high-lateral ST elevation myocardial infarction while being treated for urinary sepsis. He had no ongoing chest pain. The electrocardiogram showed sinus rhythm with a premature atrial contraction (PAC), a negative T wave in aVL, and a flattened T wave in lead I, and cardiac troponin was 4850 ng/L. Transthoracic echocardiography showed regional wall-motion abnormalities and a left ventricular ejection fraction of 45% to 50%. Coronary angiography on day 3 demonstrated a left anterior descending artery arising from the right coronary ostial region with a severe proximal stenosis and a left circumflex artery arising separately from the right coronary sinus with a severe proximal stenosis. The right coronary artery had no significant stenosis. After clinical stabilization and heart team review, elective percutaneous coronary intervention of the proximal left circumflex artery was performed 34 days after diagnostic angiography. A 3.5 x 28 mm everolimus-eluting stent was implanted, and the procedural report documented no significant residual stenosis and final Thrombolysis in Myocardial Infarction grade 3 flow. Intervention on the anomalous left anterior descending artery was deferred pending coronary computed tomography angiography. This case separates the management of a severe atherosclerotic lesion from risk assessment of an anomalous coronary origin. Invasive angiography did not establish the proximal course of the left anterior descending artery, and an interarterial or intramural course cannot be inferred from its right-sided origin. Because no acute thrombus, pre-intervention flow grade, or definitive culprit lesion was documented, the circumflex artery should be described as the presumed infarct-related artery and the delayed procedure as staged revascularization. Further management of the left anterior descending artery requires cross-sectional anatomical imaging and individualized assessment of ischemia, viability, functional status, and procedural risk.

## Introduction

Congenital coronary anomalies are uncommon, with reported prevalence varying according to the population studied and the imaging method used [1-3]. Anomalous origin of the left anterior descending artery or left circumflex artery from the right coronary sinus was identified in 0.07% of patients in one coronary computed tomography series [2]. The clinical risk of an anomalous aortic origin is determined mainly by the proximal course and by features such as an intramural segment, a slit-like ostium, an acute take-off angle, and proximal narrowing [3-7]. The association with myocardial ischemia and sudden cardiac death is best established in younger individuals with high-risk anatomy, particularly during exertion [4].

Acute myocardial infarction in a patient with anomalous coronary origins poses two separate questions: which vessel caused the acute event, and whether the congenital anatomy itself requires correction. We report a late-presenting myocardial infarction in an older, functionally dependent patient with separate anomalous right-sided origins of the left anterior descending and left circumflex arteries, multivessel atherosclerotic disease, and staged percutaneous coronary intervention of the proximal left circumflex artery.

## Case presentation

Patient information

A 79-year-old man was admitted to the coronary care unit with a diagnosis of late-presenting ST-elevation myocardial infarction while receiving treatment for urinary sepsis. He reported no ongoing chest pain. No previous cardiovascular disease was documented, and cardiovascular risk factors were incompletely characterized in the available records. A recent hospitalization for hypoxemic coronavirus disease 2019 pneumonia had been followed by marked functional decline, and he was wheelchair-dependent at presentation. His recorded medications were urapidil 30 mg twice daily, pantoprazole 20 mg once daily, and extended-release alfuzosin 10 mg once daily. The timeline of the clinical course is summarized in Table 1.

**Table 1:** Timeline of clinical events

Time	Clinical event
About 1 month before admission	An inferolateral wall-motion abnormality was reportedly present on a previous echocardiogram, limiting the ability of later echocardiography to date the infarction.
Day 0	Admission during treatment for urinary sepsis. No ongoing chest pain. Electrocardiography and troponin findings led to a diagnosis of late-presenting high-lateral ST-elevation myocardial infarction.
Day 3	Diagnostic coronary angiography identified anomalous right-sided origins of the left anterior descending and left circumflex arteries and severe proximal stenoses in both vessels.
Day 37	Elective percutaneous coronary intervention of the proximal left circumflex artery was performed 34 days after diagnostic angiography.
At manuscript preparation	Coronary computed tomography angiography and longer-term post-intervention follow-up were not available.

Clinical findings and diagnostic assessment

On admission, blood pressure was 109/72 mmHg, heart rate was 97 beats/min, and oxygen saturation was 94% on 6 L/min of oxygen. Bibasal crackles and bilateral ankle edema were present; heart sounds were regular without a murmur or rub.

Electrocardiography showed sinus rhythm with a premature atrial contraction (PAC), a negative T wave in aVL, and a flattened T wave in lead I (Figure 1). Cardiac troponin was 4850 ng/L; serial values and the assay-specific upper reference limit were unavailable.

**Figure 1:**
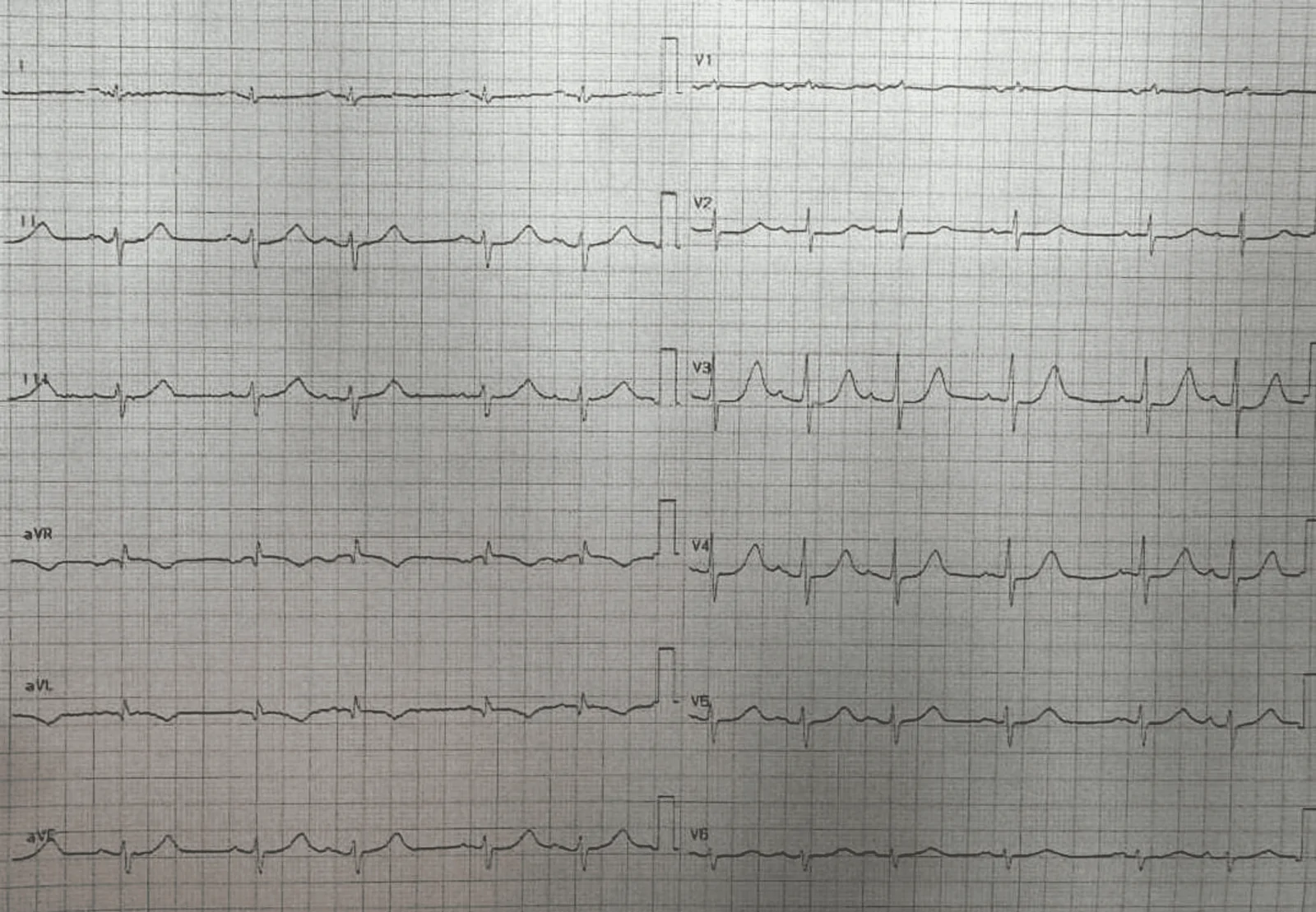
Twelve-lead electrocardiogram showing sinus rhythm with a premature atrial contraction (PAC), a negative T wave in aVL, and a flattened T wave in lead I.

Transthoracic echocardiography showed inferobasal and basal-to-mid inferolateral akinesia, mid-to-apical anterolateral hypokinesia, and a left ventricular ejection fraction of 45%-50%. Right ventricular function was preserved, with no significant valvular disease or pericardial effusion. An inferolateral wall-motion abnormality had been reported approximately one month earlier, so the examination could not date the acute event.

Coronary angiography

Diagnostic coronary angiography was performed through right radial access three days after admission. The left anterior descending artery arose from the right coronary ostial region and had an angiographically severe proximal stenosis, visually estimated at 70% to 90% (Figure 2A, 2B). The left circumflex artery arose separately from the right coronary sinus and also had a severe proximal stenosis, visually estimated at 70% to 90%. The first obtuse marginal branch had a 50%-70% stenosis, and the ostium of the second obtuse marginal branch had a 50%-70% type B1 stenosis (Figure 3). The right coronary artery arose from the right coronary sinus and had no significant stenosis.

Angiography therefore demonstrated atherosclerotic disease involving the left anterior descending and left circumflex systems together with separate anomalous origins from the right sinus region. It did not reliably define the proximal course of the left anterior descending artery. Coronary computed tomography angiography was planned to determine its relationship to the aorta and pulmonary trunk and to assess ostial and intramural features. A multidisciplinary heart team review was arranged before further revascularization.

**Figure 2:**
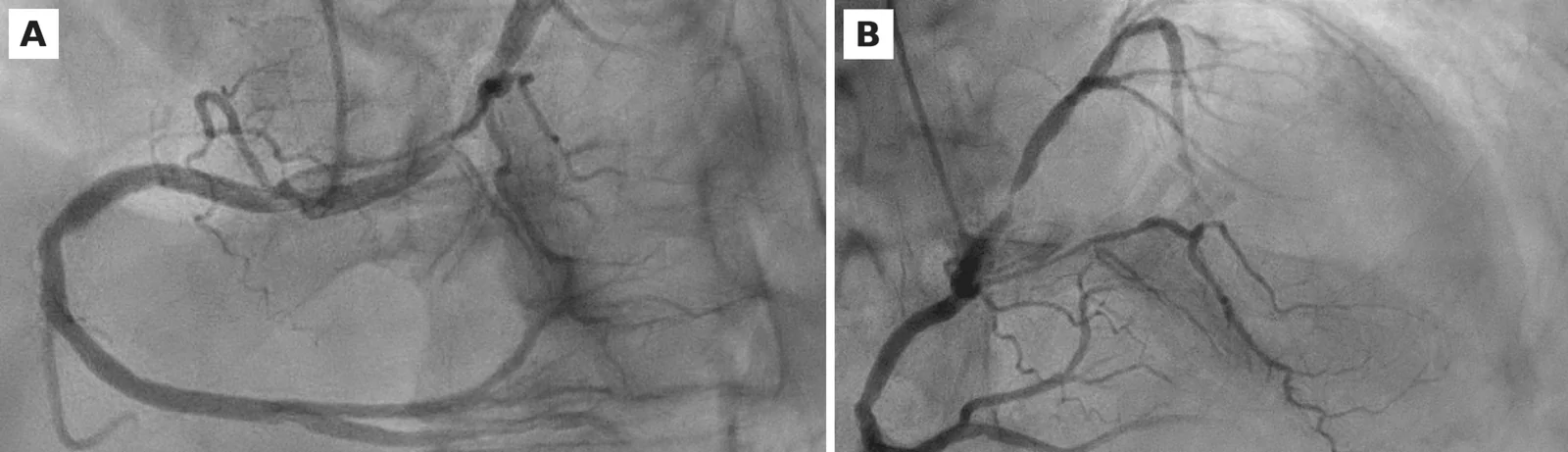
Diagnostic coronary angiography in left anterior oblique projections showing the anomalous left anterior descending artery arising from the right coronary ostial region and the severe proximal stenosis. Panel A was acquired at 32° with 7° cranial angulation; panel B was acquired at 24° with 36° cranial angulation. LAD: left anterior descending artery, RCA: right coronary artery.

**Figure 3:**
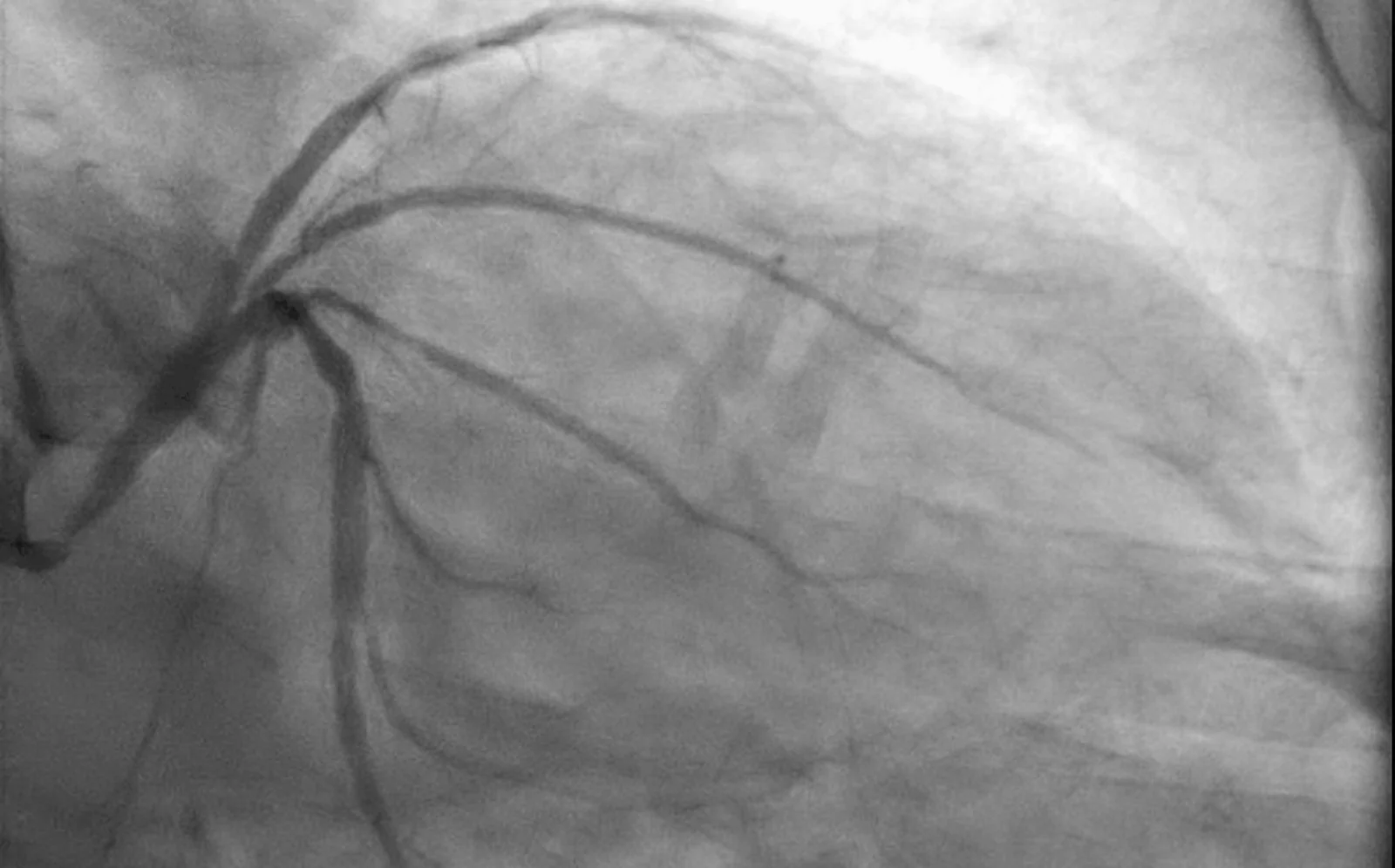
Diagnostic coronary angiography in a right anterior oblique 6° projection with 33° caudal angulation showing the anomalous left circumflex artery arising separately from the right coronary sinus and the severe proximal stenosis. LCx: left circumflex artery.

Therapeutic intervention

After treatment of the concurrent infection, clinical stabilization, and heart team review, elective percutaneous coronary intervention of the proximal left circumflex artery was performed 34 days after diagnostic angiography. The patient was asymptomatic and had no clinical heart failure at the time of the procedure. The procedural report stated that additional testing had documented silent myocardial ischemia, but the test type and results could not be identified in the available records. Treatment of the anomalous left anterior descending artery was deferred pending anatomical characterization.

The procedure was performed through right radial access with a 6-French system. A multipurpose 6-French guide catheter was positioned with difficulty because of the anomalous circumflex origin but ultimately provided adequate support. A 190-cm J-tip guidewire crossed the proximal stenosis and was advanced beyond the first obtuse marginal branch. A 3.5 x 28 mm biodegradable-polymer everolimus-eluting stent was deployed directly in the proximal left circumflex artery at 14 atm for 20 seconds. The procedural report documented no significant residual stenosis and final Thrombolysis in Myocardial Infarction grade 3 flow. Representative procedural views are shown in Figure 4A and Figure 4B. Dual antiplatelet therapy was recommended for 6 to 12 months.

**Figure 4:**
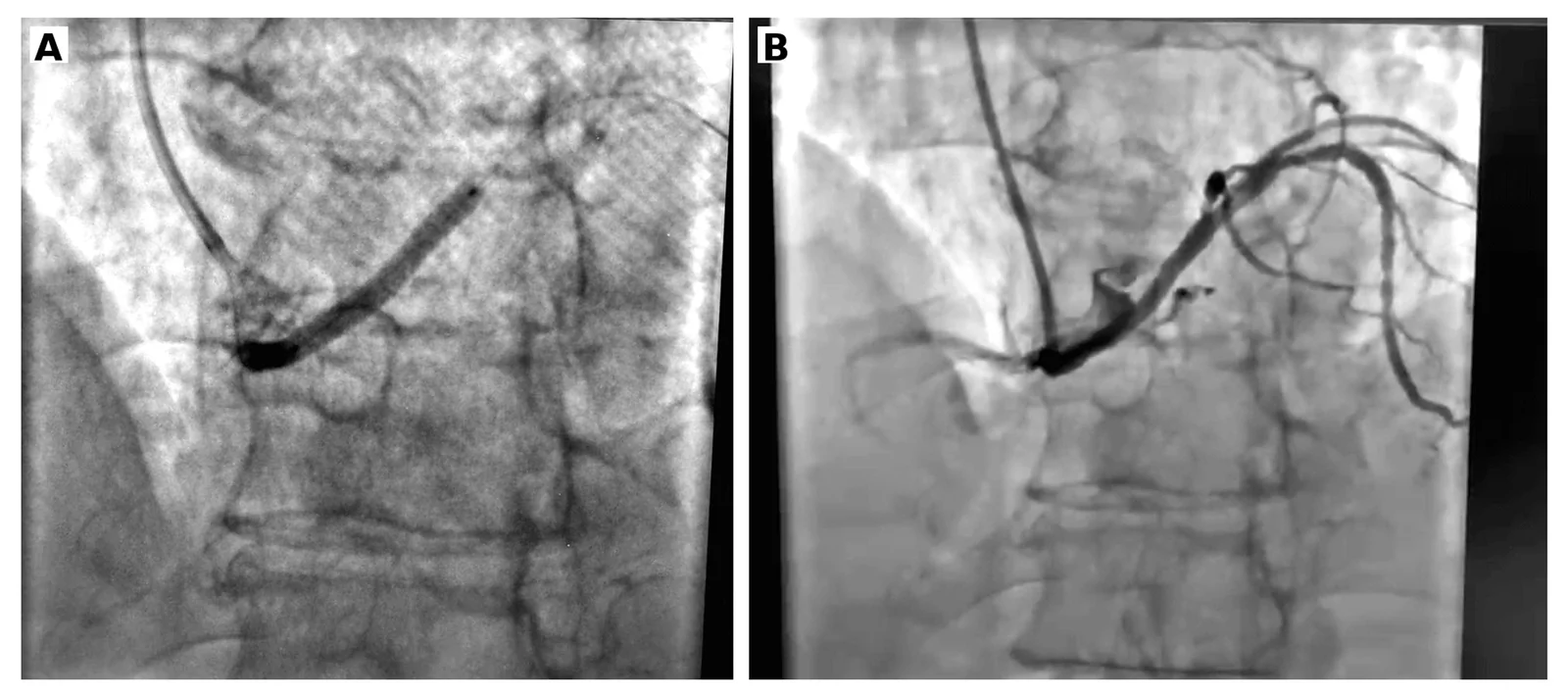
Representative procedural angiographic views from the staged percutaneous coronary intervention of the proximal left circumflex artery. (A) Stent deployment across the proximal left circumflex artery stenosis. (B) Angiographic result after stent implantation, showing good restoration of the coronary lumen with no significant residual stenosis.

Follow-up and outcomes

Coronary computed tomography angiography had not been performed at the time of manuscript preparation. The exact proximal course of the left anterior descending artery, ostial morphology, and presence or absence of an intramural segment therefore remained unknown. Longer-term post-intervention clinical follow-up was not available in the records reviewed for this report.

## Discussion

The central finding in this case is the coexistence of two distinct problems: severe atherosclerotic disease and anomalous right-sided origins of both major left coronary branches. Diagnostic angiography established the origins and stenoses but did not establish the proximal course of the left anterior descending artery. This distinction is important because an anomalous origin from the opposite sinus is not inherently equivalent to an interarterial or intramural course [3,5,7].

Anatomical risk assessment

Coronary computed tomography angiography is the preferred non-invasive method for defining the coronary origin, proximal course, and relationship to the great vessels. It can also identify an intramural segment, a slit-like orifice, an acute take-off angle, an elliptical proximal lumen, and focal proximal narrowing [6,7]. Cardiac magnetic resonance can add information on ventricular function, scar, perfusion, and viability. In this patient, the left circumflex artery would usually be expected to follow a retroaortic course, which is generally regarded as lower risk, whereas the left anterior descending artery could follow an interarterial, intramural, prepulmonic, retroaortic, or intraseptal route [7-8]. None of these courses can be assigned confidently from the available still angiographic images.

The evidence linking anomalous coronary origins to exertional sudden death comes largely from younger populations [4]. It should not be extrapolated directly to a 79-year-old patient with severe fixed atherosclerotic stenoses and major functional impairment. Anatomical findings remain relevant, but age, symptoms, ischemic burden, ventricular function, frailty, life expectancy, and patient preference should determine the management strategy [1-7].

Myocardial infarction and culprit attribution

The clinical record classified the presentation as a late high-lateral ST-elevation myocardial infarction. The electrocardiographic changes, troponin elevation, regional wall-motion abnormalities, and obstructive coronary disease support an ischemic diagnosis. However, the patient also had urinary sepsis, which can cause acute non-ischemic myocardial injury or secondary myocardial infarction. Current diagnostic criteria require acute myocardial injury, usually demonstrated by a rise or fall in cardiac troponin, together with evidence of acute myocardial ischemia [9]. The absence of serial troponin values, the assay-specific upper reference limit, a clearly new wall-motion abnormality, acute thrombus, and a documented pre-intervention flow grade prevents definitive classification of the infarct mechanism from the available records.

The left circumflex artery was considered the presumed infarct-related artery because the high-lateral electrocardiographic pattern and inferolateral dysfunction were anatomically compatible with its territory and because a severe proximal stenosis was present. Nevertheless, the inferolateral wall-motion abnormality had reportedly been present approximately one month earlier, and no acute circumflex occlusion was documented. Culprit attribution should therefore remain qualified. The intervention performed 34 days after diagnostic angiography was an elective staged procedure rather than immediate reperfusion. This wording is consistent with its timing, the absence of ongoing chest pain or instability, and the heart team decision after treatment of the concurrent infection [10].

Revascularization strategy

Percutaneous intervention in anomalous coronary anatomy can be limited by non-coaxial guide engagement, inadequate support, an acute take-off angle, and uncertain ostial geometry [11]. In this case, an MP1 guide catheter provided sufficient support for direct stenting of the proximal left circumflex lesion. The procedural report documented an acceptable final angiographic result, although the available still images could not independently confirm their timing or the final flow grade.

Further intervention on the left anterior descending artery should await cross-sectional anatomical characterization. If coronary computed tomography angiography identifies high-risk anatomy, stress imaging or invasive physiological assessment may help determine whether ischemia is attributable to the anomalous segment, the fixed atherosclerotic stenosis, or both. Surgical repair is most clearly considered when high-risk anatomy is accompanied by symptoms or objective ischemia, but the operation must be matched to the anatomy. Unroofing is used for a suitable intramural segment, whereas reimplantation, ostial reconstruction, pulmonary artery translocation, or bypass grafting may be considered in selected configurations [1-7]. Additional surgical outcome data [12] and evidence for protocolized unroofing [13] may inform procedure selection. In this older, functionally dependent patient, optimal medical therapy, percutaneous treatment, surgery, and surveillance should be compared through shared decision-making after formal frailty and operative-risk assessment.

Limitations

This report has important limitations. Coronary computed tomography angiography was unavailable, so the proximal course of the left anterior descending artery and other high-risk anatomical features could not be fully characterized. The exact timing of symptom onset and infarction could not be established, and serial troponin values, the troponin assay and assay-specific upper reference limit, the presence or absence of an acute thrombus, and the pre-intervention flow grade were not documented. The test reportedly demonstrating silent ischemia could not be identified. The available records did not provide complete details of the discharge medications, the final duration of dual antiplatelet therapy, or longer-term clinical follow-up. These limitations prevent definitive attribution of the culprit vessel and limit characterization of the congenital coronary anomaly and assessment of the long-term clinical outcome.

## Conclusions

A severe proximal left circumflex stenosis was successfully treated by elective staged percutaneous coronary intervention in an older patient with late-presenting myocardial infarction and anomalous right-sided origins of the left anterior descending and circumflex arteries. The right-sided origin of the left anterior descending artery alone did not establish its proximal course or the presence of high-risk anatomical features, for which coronary computed tomography angiography would be required. Further management of the anomalous left anterior descending artery should therefore be guided by anatomical characterization and, when appropriate, functional assessment. Culprit-vessel attribution and the distinction between acute reperfusion and later planned revascularization should remain explicit when supporting acute-phase data are incomplete.
